# Injections into the Bronchial Tubes and into Tubercular Cavities, of Solution of Nitrate of Silver

**Published:** 1855-10

**Authors:** 


*Injections into the Bronchial Tubes and into Tubercular Cavities, of Solution of Nitrate of Silver.*—Dr. Horace Green presented to the New York Academy of Medicine, in December last a communication in which he claimed to have treated bronchial affections and tuberculosis with advantage, by the direct introduction into the lungs of a strong solution of nitrate of silver, injected through an elastic tube. The subject was referred to a committee, to investigate and report on the subject.

At the meeting of the Academy, on Wednesday, June 6th, Dr. Willard Parker, the eminent Professor of Surgery in the College of Physicians and Surgeons, who was chairman of the committee, made a report on behalf of a majority of that committee. We take the following summary of the report from the New York *Daily Times* of June 11th.

Dr. Parker stated that the Committee had held five meetings—two at the office of Dr. Green, three at Bellevue Hospital, and had experimented upon thirty-eight individuals, and would now present, 1. A History of catheterism of the air passages; 2. The results of their experiments; and 3. The opinions founded upon these experiments.

1. Catheterization of the air passage is no new idea: it is recommended by Hippocrates; and Ryland, in his work on Diseases of the Larynx, devotes a short chapter to it.

2. The result of their experimentation proved, to the satisfaction of the whole Committee, that the operation is possible. In eleven cases the instrument entered without doubt into the trachea. This was evident to the finger of the operator by local examination. Moreover the symptoms manifested by the patient are unequivocal. They are such as always accompany the entrance and the presence of a foreign body in the air passage, spasm, suffusion of eyes, redness of face, a sense of suffocation, and a desire to withdraw the instrument.

In most instances the instrument passed into the œsophagus, and the report next gave a tabular statement of unequivocal rational signs by which it might be determined into which passage the instrument was passed. [This tabular statement is very incomplete.]

When the instrument is passed into the trachea, the face becomes turgid, respiration croupy, there is spasmodic cough, and a sense of immediate impending suffocation. If assured that there is no danger (in one instance seen), the patient can be enabled to bear the instrument for a short period.

When the instrument enters the œsophagus there is a hoarse, stridulous voice, retching, and cough, generally vomiting; but after a short interval these symptoms pass away: the irritation is allayed, and the voice becomes nearly natural. In but one case were these peculiarities absent, and in that sensibility of the parts was deadened by previous syphilitic disease.

As a rule the rational signs will distinguish the location of the instrument. The blowing out of the candle by expiration through the tube, and the inflation of a bladder tied over the end, are not sound tests, as this might be done by the emission of gas from the stomach, when the tube was purposely passed into the œsophagus.

The opinion of the operator is unreliable when based upon his own opinion, without reference to these rational signs. Dr. Green being himself repeatedly mistaken, as confessed by him.

The sensations of patients are often reliable after many applications have taught the different feelings.

The facility of the operation does not depend upon the previous preparation of the patient, as insisted on by Dr. Green, for, with the exception of Wetmore, patients who had frequent applications of the caustic to the fauces for six months were equally intolerant as the new patients.

Much depends upon the character and form of the instruments. With a tube curved by a stylet to a form corresponding to a circle of six inches diameter, in thirteen cases five failed, or 38 per cent. of failure, which proves that the trachea may be entered with a very considerable certainty.

With a tube with a small curve, such as used by Dr. Green, in 38 cases 35 failed or 92 per cent. of failure.

With a sponge probang, in 18 cases there were 18 failures, or 100 per cent. of failure.

The conclusion is, that the sponge probang, or slightly curved tube, cannot be made to enter the trachea, if largely curved, can; that local application within the trachea is difficult, and rarely successful; and whether an instrument may be passed at will into the right or left bronchi, the committee leave to the Academy to decide from these facts.

The committee considered themselves excused from entering into the therapeutical value of the application, as proposed on the grounds that they do not think it advisable to discuss a mere theoretical question. A post-mortem of a consumptive is given, where death followed in twenty-six hours after operation.

This report was signed by Dr. Parker, I. Wood, and H. O. Stone. Dr. A. H. Stevens is absent from the city, and two other members reported to concur in it, but they were absent and their names were not appended.

Prof. Baker, the colleague of Dr. Green in the New York Medical College, made a minority report, stating the reason why he could not sign the majority report.

At an adjourned meeting of the academy, on the following Wednesday, the reports were again read, and after some debate the reports and the paper of Dr. Green were ordered to the committee on publication, to be printed in the *Transactions* of the Academy, and distributed to the members previous to the next meeting.

The Society then adjourned to the next regular meeting on the second Wednesday in July.—*Medical News.*

